# Developmental, cytogenetic and epigenetic consequences of removing complex proteins and adding melatonin during in vitro maturation of bovine oocytes

**DOI:** 10.3389/fendo.2023.1280847

**Published:** 2023-10-23

**Authors:** Desmond A. R. Tutt, Gizem Guven-Ates, Wing Yee Kwong, Rob Simmons, Fei Sang, Giuseppe Silvestri, Carla Canedo-Ribeiro, Alan H. Handyside, Remi Labrecque, Marc-André Sirard, Richard D. Emes, Darren K. Griffin, Kevin D. Sinclair

**Affiliations:** ^1^ School of Biosciences, University of Nottingham, Sutton Bonington, United Kingdom; ^2^ School of Veterinary Medicine and Science, University of Nottingham, Sutton Bonington, United Kingdom; ^3^ Paragon Veterinary Group, Carlisle, United Kingdom; ^4^ School of Life Sciences, University of Nottingham, Nottingham, United Kingdom; ^5^ School of Biosciences, University of Kent, Canterbury, United Kingdom; ^6^ L’Alliance Boviteq Inc., Saint-Hyacinthe, QC, Canada; ^7^ CRDSI, Département des Sciences Animales, Faculté des sciences de l’agriculture et de l’alimentation, Université Laval, Quebec City, QC, Canada

**Keywords:** oocyte, aneuploidy, DNA methylation, IVM, serum, melatonin, cumulus cells, blastocyst

## Abstract

**Background:**

*In vitro* maturation (IVM) of germinal vesicle intact oocytes prior to *in vitro* fertilization (IVF) is practiced widely in animals. In human assisted reproduction it is generally reserved for fertility preservation or where ovarian stimulation is contraindicated. Standard practice incorporates complex proteins (CP), in the form of serum and/or albumin, into IVM media to mimic the ovarian follicle environment. However, the undefined nature of CP, together with batch variation and ethical concerns regarding their origin, necessitate the development of more defined formulations. A known component of follicular fluid, melatonin, has multifaceted roles including that of a metabolic regulator and antioxidant. In certain circumstances it can enhance oocyte maturation. At this stage in development, the germinal-vesicle intact oocyte is prone to aneuploidy and epigenetic dysregulation.

**Objectives:**

To determine the developmental, cytogenetic and epigenetic consequences of removing CP and including melatonin during bovine IVM.

**Materials and methods:**

The study comprised a 2 x 2 factorial arrangement comparing (i) the inclusion or exclusion of CP, and (ii) the addition (100 nM) or omission of melatonin, during IVM. Cumulus-oocyte complexes (COCs) were retrieved from stimulated cycles. Following IVM and IVF, putative zygotes were cultured to Day 8 in standard media. RNAseq was performed on isolated cumulus cells, cytogenetic analyses (SNP-based algorithms) on isolated trophectoderm cells, and DNA methylation analysis (reduced representation bisulfite sequencing) on isolated cells of the inner-cell mass.

**Results:**

Removal of CP during IVM led to modest reductions in blastocyst development, whilst added melatonin was beneficial in the presence but detrimental in the absence of CP. The composition of IVM media did not affect the nature or incidence of chromosomal abnormalities but cumulus-cell transcript expression indicated altered metabolism (primarily lipid) in COCs. These effects preceded the establishment of distinct metabolic and epigenetic signatures several days later in expanded and hatching blastocysts.

**Conclusions:**

These findings highlight the importance of lipid, particularly sterol, metabolism by the COC during IVM. They lay the foundation for future studies that seek to develop chemically defined systems of IVM for the generation of transferrable embryos that are both cytogenetically and epigenetically normal.

## 1 Introduction

*In vitro* maturation (IVM) of germinal vesicle (GV) intact oocytes prior to *in vitro* fertilization (IVF) is practiced commonly in livestock species (1). In cattle, GV oocytes are collected either from abattoir derived ovaries (a by-product of meat production) or by transvaginal follicular aspiration (ovum pickup; OPU) from living donors (2, 3). Historically, IVM has proven to be technically more challenging in human assisted reproduction (ART) (4) and so is practiced less widely; the procedure being used mostly for fertility preservation or where ovarian stimulation is contraindicated (5–7). However, recent advances in oocyte recovery and IVM culture methods have meant that IVM is beginning to gain traction in human ART (8, 9).

During IVM, GV oocytes, arrested at the diplotene stage of prophase I, undergo meiotic resumption and transit towards metaphase II prior to IVF (10). In normal ovarian cycles leading to spontaneous ovulation, these cytogenetic events coincide with a carefully choreographed series of cytoplasmic and molecular modifications within the oocyte, as well as molecular/metabolic exchanges between the oocyte and surrounding follicular cells. Such events occur over several days and ultimately determine the post-fertilization developmental competency of the egg (11–13). Follicular aspiration for recovery of GV oocytes and IVM truncates these processes and subjects the maturing oocyte to suboptimal physiological conditions that currently represent a poor facsimile of the natural environment offered by the ovarian follicle.

For the most part, commonly employed protocols rely on relatively basic media for both livestock (2) and human (5) IVM. These protocols generally utilize readily available commercial base medium (2, 14) and include FSH to promote oocyte maturation (15). However, the follicle-enclosed oocyte that matures *in vivo* is exposed, naturally, to a more complex and dynamic microenvironment comprising a myriad of stimuli from hormones, growth factors, lipids, antioxidants and other metabolites (16–22). These arise through interactions with surrounding fluids, resident cumulus, granulosa and theca cells (23, 24). This reliance on multiple interactions, along with the oocyte’s changing metabolic requirements during maturation, renders the formulation of a physiologically relevant and effective IVM medium particularly challenging.

As a substitute for the complex interplay with follicular fluid components, undefined biological protein complexes are frequently added to IVM media. In the case of livestock IVM, this primarily comes in the form of fetal calf serum and/or albumin (25, 26) and, to a lesser extent, follicular fluid (27). Human IVM on the other hand has relied predominantly on maternal serum and/or albumin, or human follicular fluid (5). The addition of such complex proteins (CP) presents several issues, not least of which relates to ethical concerns regarding their origin (28). Their undefined nature and biological variation between batches contribute to poorly reproducible results (29). Therefore, removal of CP from IVM media can be considered a necessary first step required to introduce future specific refinements to this stage of *in vitro* embryo production (IVP). As a biological material, CP also confer a risk of disease transmission (30), and there are concerns relating to potential adverse epigenetic effects on offspring such as, but not limited to, those associated with Large Offspring Syndrome reported in livestock (31, 32).

A component of follicular fluid with a myriad of effects on the follicular environment is melatonin, which interacts with thecal (33), granulosa (34, 35) and cumulus (36, 37) cells, together with the oocyte itself (38, 39). Levels of melatonin in follicular fluid are linked to oocyte developmental capacity (40, 41). Specifically, the addition of melatonin to livestock IVM media in the presence of CP can reduce reactive oxygen species (ROS) (38, 42). It can alter metabolism, particularly lipid utilization and storage (43, 44), leading to improved oocyte maturation (38, 45) and embryo development (45, 46). Improvements in oocyte and embryo development have also been reported when melatonin is included in human IVM media (47, 48). Here, melatonin is of particular interest in cases of PCOS, given that abnormally low concentrations of this indolamine in follicular fluid are a characteristic feature of PCOS (49, 50). Indeed, the addition of melatonin to IVM media improved embryo implantation rates for oocytes retrieved from PCOS patients (51). However, melatonin effects during cell culture are modified in the absence of CP (52, 53), but how such interactions influence IVM is not yet known.

The foregoing discussion highlights the need to develop more chemically defined systems of IVM for mammalian GV-stage oocytes that limit or eliminate the use of CP. Moreover, we need to understand how this may modify the effects of remaining IVM components. With these thoughts in mind, the current article reports on the first of a series of ongoing experiments that seek to develop more chemically defined systems of IVM for the laboratory production of embryos (IVP). Two aspects are considered: the first relates to the complete removal of CP from our standard IVM media (54, 55) to be replaced with polyvinylpyrrolidone (PVP) as a macromolecule. The rationale was to determine how well oocytes would mature, and embryos develop, following IVF under these minimal conditions. The cytogenetic and epigenetic status of advanced (potentially transferable) blastocysts was also of interest as we previously reported that these can be affected by IVM in our culture system (55, 56), and may be predictive of pregnancy outcomes upon embryo transfer (57, 58). The second aspect considered the developmental, molecular and metabolic effects of adding melatonin to both CP containing media (which we hypothesized would be beneficial), and to defined media (i.e., with PVP), for which there is limited information. We report (i) the potential to generate modest yields of transferrable quality embryos in the absence of CP; (ii) beneficial effects of melatonin in the presence but detrimental effects in the absence of CP; and (iii) no effect of IVM media composition on the incidence of chromosomal abnormalities but altered metabolism (primarily lipid) in cumulus-oocyte-complexes (COC) which manifests as distinct metabolic and epigenetic signatures several days later in expanded and hatching blastocysts. These findings highlight the importance of lipid metabolism by the COC during IVM and lay the foundation for future studies that seek to develop defined systems of IVM that lead to the generation of transferrable embryos which are both cytogenetically and epigenetically normal.

## 2 Materials and methods

### 2.1 Generic considerations

All animal procedures adhered to the Animals (Scientific Procedures) Act, 1986. Associated protocols complied with the ARRIVE guidelines and were approved by the University of Nottingham Animal Welfare and Ethical Review Body (AWERB) with project licensed authority (PDBF3E539; 29/05/2019). All chemicals and reagents were sourced through Sigma-Aldrich Company Ltd (Dorset, UK) unless otherwise specified.

### 2.2 Experimental design, animals, estrous synchronization and ovarian stimulation

Cumulus-oocyte complexes (COCs) utilized in this study were retrieved from four cycles of ultrasound guided, transvaginal follicular aspiration (Ovum Pick-Up; OPU) involving eight 13-16 month-old post-pubertal Holstein-Friesian heifers. These animals were bred and accommodated at the University of Nottingham dairy farm and fed a standard grass/maize silage-based diet formulated to meet the nutrient requirements of young heifers growing at around 0.8 kg/d (59).

Heifers were paired at random for the purposes of COC allocation to each of four *in vitro* maturation (IVM) treatment groups. This allocation was rotated at the end of each of the four cycles so that oocytes from each donor were allocated to each of the four treatments during the study. Oocytes were matured, fertilized, and zygotes cultured to the blastocyst stage whilst retaining individual donor identity. This involved using separate maturation vials and culture wells for each donor. The study consisted of a 2 x 2 factorial arrangement which compared (i) the inclusion (+CP) or exclusion (-CP) of complex proteins (derived from serum and albumin) and (ii) the addition (+M; 100 nM) or omission (-M) of melatonin, both during *in vitro* maturation (IVM).

Estrous cycles were synchronized initially by insertion of an intravaginal progesterone device (PRID^®^ Delta, CEVA Santé Animale, Libourne, France; impregnated with 1.55g P4) and 125 µg GnRH i.m. (Acegon, Zoetis UK Ltd, Leatherhead, UK) (both administered on Day -12), followed by 150 µg prostaglandin i.m. (Prelim, Zoetis UK Ltd, Leatherhead, UK) administered on Day -6, and PRID^®^ Delta withdrawal and a second prostaglandin injection (both on Day -5). A second GnRH injection was then administered on Day -4, and ablation (aspiration) of all follicles ≥ 5 mm in diameter (dominant follicle removal; DFR) was undertaken on Day 0. Each heifer received a PRID^®^ Delta following DFR and ovarian stimulation commenced 48h later. This involved six injections (i.m.) of follicle stimulating hormone (FSH; Folltropin, 70IU dose per injection, Vetoquinol UK Ltd, Towcester, UK) given at 12 h intervals. The first session of OPU was undertaken approximately 38-42 h following the final FSH injection. All OPU procedures were undertaken in a dedicated theatre where the ambient temperature was maintained between 30 and 34°C. Following OPU, a replacement PRID^®^ Delta was inserted and the subsequent cycle of DFR commenced eight days later.

### 2.3 Collection and grading of COCs

Cumulus-oocytes complexes were aspirated as described previously (55, 60). Briefly, OPU used a Cook Medical vacuum pump with a 7.5 MHz ultrasound scanner (Exapad, IMV Imaging, Glasgow, UK) with aspiration pressure set at -70 mmHg. COCs were aspirated through an 18G needle and 1.4 m of 1.4 mm (I.D.) silicone tubing into 5 mL of Tyrodes lactate-based aspiration media, as described previously (60). OPU aspirants were passed through a heated (~37°C) filter, and filtrates transferred to 100 mm petri dishes on a heated stage (~38°C) for COC retrieval. COCs were graded 1-4 according to (61, 62). All COCs with sparse, expanded or absent cumulus or with fragmented, pale or irregular cytoplasm were classed grade 4 and rejected.

### 2.4 *In vitro* embryo production

Grade 1 COCs were trimmed using an 18-G needle to approximately 5 layers of cumulus cells (CCs). Removed CCs were collected into Ca^2+/^Mg^2+^ free PBS/0.1% PVP (PBS/PVP) on ice, and later washed in PBS/PVP, pelleted (by centrifugation) and stored at -80°C.

Base media for oocyte maturation was HEPES buffered TCM199 supplemented with 0.2 mM pyruvate, 50 µg/mL gentamicin, 5 µg/mL FSH, 0.5 µg/mL LH (Lutropin-v, Bioniche Animal Health), 1 µg/mL E2, and 2.5 mM L-carnitine [as described previously (54)]. Base media was further modified to create the four aforementioned-treatment groups. Media containing ‘Complex Proteins’ (+CP) included 10% (v/v) FBS [Gibco (10082139)] and 4 mg/mL fatty acid free BSA (MP Biomedicals (9048-46-8) California, USA). In contrast, ‘Defined Media’ (-CP) included 4 mg/mL polyvinylpyrrolidone (average mol wt. 40,000; PVP40) as a substitute for CP. Melatonin (Sigma-Aldrich; M5250, Lot SLCC7825) was added at 100nM in line with previous studies that reported positive effects at this concentration during IVM and IVC (39, 42). Thus, the four IVM treatment combinations were: Complex Proteins (+CP-M), CP plus Melatonin (+CP+M), Defined Media (-CP-M), and Defined Media plus Melatonin (-CP+M). Oocyte maturation was completed in screw top cryovials (Thermo Fisher Scientific Inc. Loughborough, UK) at atmospheric CO_2_ and 38.5°C for 23-24 h (55, 60).

Following maturation, oocytes were gently drawn into a fine-bore glass pipette to remove expanded CCs, leaving the corona radiata intact. Cumulus cells were washed through 50 µl drops of PBS/PVP and collected into PBS/PVP on ice and later pelleted and stored at -80°C. Oocytes were transferred to Tyrodes lactate-based fertilization media and inseminated with gradient purified sperm from a single sire (55, 60). Briefly, frozen-thawed semen from a single bull was prepared by centrifugation through a 45%/90% gradient (BoviPure; Nidacon International AB, Mölndal, Sweden). Fertilization occurred in 50 μL drops (maximum of 5 oocytes per drop) of modified Tyrode’s lactate media under oil (final concentration of 70,000 sperm per drop). These gametes were co-cultured for 18-21 h in a humified environment of 5% CO_2_ in air at 38.5°C. Resultant zygotes were cultured in SOF based sequential media in drops under oil maintained in a humidified environment at 6.8% CO_2_, 5% O_2_ and 38.5°C, with media changed at 72, 120 and 168 h (55). Embryos were cultured at no more than 11 per 10 μL drop for the first two media changes, and then transferred to 20 μL drops (Day 6) following the final media change. Embryo development was assessed at 48 and 120 h and blastocysts assessed for stage and quality in accordance with the International Embryo Transfer Society (IETS) guidelines for bovine embryo assessment (63) at 168 h. The most advanced blastocysts (up to 4/donor/cycle) were immuno-dissected (55), and inner cell mass (ICM) and trophectoderm (TE) samples frozen individually in 4 µl PBS at -80°C for later DNA methylation (ICM) or karyotype/mtDNA (TE) analyses. All remaining blastocysts were pooled (by donor) in 4 µl PBS, and frozen at -80°C. Spent media drops were collected and pooled by treatment, and frozen at -80°C for metabolic analyses.

### 2.5 RNAseq analyses of cumulus cells

Cumulus cell pellets were thawed, cells lysed and RNA extracted using PicoPure RNA Isolation Kit (Thermo Fisher Scientific Inc.) as per manufacturer’s instructions for cell pellets. For each sample cDNA was generated from 100ng of total RNA using the QuantSeq 3’ mRNA-Seq library prep kit for Illumina (FWD) (Lexogen GmbH, Vienna, Austria) following the manufacturer’s instructions. Libraries were then sequenced on the Illumina NextSeq 500 (Illumina, San Diego, USA) using a NextSeq 500 High Output v2.5 75 cycle kit (Illumina) to generate approximately 5 million 75bp single-end reads per library.

Raw reads were aligned to the *Bos taurus* ARS_UCD1.2 reference genome, using the Bluebee Genomics Analysis Platform (https://www.bluebee.com/lexogen) according to the manufacturer’s instructions, with unique and correctly aligned reads being taken into the counts file. DESeq2 (version: 1.24.0) was used to detect the differentially expressed genes for each comparison using default settings. Gene enrichment analysis used ShinyGo v0.76.3 (http://bioinformatics.sdstate.edu/go/) (64) with a minimum pathway size of 2 and FDR cutoff of 0.05 to identify pathways enriched for genes differentially expressed between treatments. Heatmaps were performed using bespoke R scripts using heatmap.2 from the gplots package v 3.0.1 (65).

### 2.6 Chromosomal errors and mitochondrial DNA copy number in the trophectoderm

Isolated TEs were employed for whole genome amplification (WGA) (55). The WGA DNA output was split for SNP array analysis (and subsequent chromosomal analysis), and for mitochondrial copy number analysis. SNP array analyses were performed by Neogen Europe Ltd (Ayr, Scotland, UK) as described previously (55), with the exception that the chip used was a GeneSeek^®^ GGP Bovine 100K SNP (Illumina, Cambridge, UK). The resultant data was used for chromosomal error analyses, as described previously (55, 58). Briefly, chromosomal abnormalities were detected by applying three PGT-A algorithms for each embryo: signal intensity data B-Allele Frequency (BAF) and Log R Ratio (LRR) graphs, Karyomapping (66), and Gabriel-Griffin plots (67). BAF and LRR were employed to detect copy number variations, whereas Karyomapping was used to investigate the parental origin (maternal or paternal) of each abnormality and for detection of triploidy and uniparental disomy. Gabriel-Griffin plots were also employed to understand the meiotic origin of trisomies (MI, MII, or mitotic). For each abnormality, mosaicism state was inferred through the LRR values.

Due to the limited number of SNPs available for chromosome Y, this chromosome was not analyzed by Karyomapping as haploblock tracing was not possible. Paternal chromosome X errors were also not characterized by Karyomapping due to the inability to perform haploblock tracing, as in the sire, sex chromosomes are present in a single copy.

Mitochondrial DNA copy number was determined by qPCR using primers for the mitochondrial gene NADH dehydrogenase subunit 4 (ND4) (F: CTCGCCTTCCTTTACACGGGA, R: GCAGTTCTTGCATACTTTTTCGGTA), normalized to a single copy-number gene (Protein arginine N-methyltransferase 7 (PRMT7)) (F: TGGCAAGGCTGCTTTTCTCT, R: ACCTTGCAATCTCCTGGTGG). PCR conditions were 95°C for 2 min, followed by 40 cycles at 95°C for 5s and 60°C for 10s, then a final cycle of 95°C for 10s, followed by a melt curve of 65°C to 95°C at 0.5 °C increments. To generate a standard curve, ND4 and PMRT7 were amplified from WGA DNA and purified using the Zymoclean gel DNA recovery kit (Zymo Research, Irvine, USA) according to the manufacturer’s instructions. PCR products were then sequenced to confirm identity prior to quantification using the Qubit HS dsDNA assay kit (Thermo Fisher Scientific Inc.). A standard curve (6 points of 5-fold serial dilution 1 x10^-5^ to 3.2 x 10^-9^ copies per reaction) was incorporated with each qPCR, and samples run in duplicate. Amplification efficiencies for both genes were >95%.

### 2.7 ICM reduced representation bisulfite sequencing

In order to generate RRBS libraries, DNA was extracted from pools of five stage-matched ICMs to form three ‘biological replicates’ across the four treatment groups. Within each ‘biological replicate’, ICMs were derived from the same four donor animals for each of the four treatments. Libraries were prepared based on (56). Briefly, extracted DNA from pooled ICMs (in 20 µl PBS within a single tube) underwent restriction enzyme (MspI) digestion, end-repair, dA tailing, adaptor ligation, and bisulfite conversion (68). Unmethylated-DNA (8 pg/µL, Promega, Southampton, UK) was added to samples prior to MSP1 digestion to monitor completeness of bisulfite conversion. Bisulfite converted DNA was then amplified by PCR with KAPA HiFi uracil+ (Roche Diagnostics Ltd, West Sussex, UK) for 22 cycles, followed by size selection of 150-600 bp amplified DNA fragments and primer adapter removal using the BluePippin system (Sage Science, Beverly, MA, USA). Final RRBS libraries (x12) were sequenced to a depth of ~80 million 150 bp paired-end reads using an Illumina NovaSeq6000 S4 platform (Novogene, Cambridge, UK).

Bioinformatic analyses: Multiplexed sequencing reads were trimmed to remove adapter sequences and low-quality bases using skewer with commands (-Q 20, -q 3) (69). Trimmed reads were aligned to the bovine reference genome (Bta.ARS-UCD1.2.97) using bisulfite read mapper Bismark (70) using default settings. Duplicate reads were marked using the MarkDuplicates module of Picard tools [https://broadinstitute.github.io/picard/] and methylation values extracted using bismark_methylation_extractor module (commands –no_overlap –paired-end). Methylation values were extracted from output SAM format files using methylKit (71) (nolap=TRUE, mincov=5, minqual=20). To avoid methylation differences being called which may be related to underlying genetic differences of cells analyzed, bases at known variant positions (as reported in Bta.ARS-UCD1.2.97) were removed. Differentially methylated cytosines (minimum difference of >10% (72–74); between groups were identified using limma (75) and annotated using the genomation package (76). Gene enrichment analysis of ‘Biological Process’ gene ontology terms was performed using GeneTrail (77) overrepresentation enrichment algorithm with a significance value of 0.05 adjusted as per (78), with a size category minimum of 2 and maximum of 700.

### 2.8 Spent media glucose and amino acid analyses

Spent and control media from two replicates were analyzed for glucose concentration. Media were thawed at room temperature and immediately diluted 1:25 in PBS and analyzed using a commercial glucose assay (GlucoseGlo, Promega, Southampton, UK) according to the manufacturer’s instructions and measured on a luminescent plate reader (Spectramax 5 – Molecular Devices, San Jose, USA). Spent and control media from three replicates were analyzed for amino acid concentration as previously described (79, 80) but modified to account for small sample volume. Briefly, media were thawed at room temperature and 24 µL used for assay. To this, 6uL solution of mixture of internal standards Norleucine (1mM) and 5-sulphosalicylic acid (60mg/mL) was added, and incubated at 4°C for 60 min. It was then centrifuged at 13000 rpm for 15 minutes. Amino acids in the top layer were then measured using a Biochrom 20+ amino acid analyzer (Biochrom Ltd, Cambridge). EZChrom Elite Software (Agilent Technologies, Inc., Santa Clara, CA) was used for peak integration.

### 2.9 Statistical analyses

Analyses were performed using the GenStat statistical package (21st Edition, VSN International, 2022; https://www.vsni.co.uk/). All data associated with embryo development were analyzed using restricted maximum likelihood (REML) generalized linear mixed models that, in the case of proportions, assumed binomial errors and used logit-link functions. In the case of counts, models assumed Poisson errors (with log-link functions) and normal errors for metabolic and molecular data. ‘Donor’ formed the random effect in these models, whereas fixed effect terms were ‘Cycle’, ‘Media’ (Complex vs Defined), ‘Melatonin’ (Present vs Absent), and interactions between these two latter terms. Data are presented as means ± SEM.

## 3 Results

Briefly, to reiterate, this study consisted of a 2 x 2 factorial arrangement that compared (i) the inclusion (+CP) or exclusion (-CP) of ‘complex proteins’ (derived from serum and albumin) and (ii) the addition (+M; 100 nM) or omission (-M) of melatonin, both during IVM. Cumulus oocyte complexes (COCs) were retrieved from stimulated (‘coasted’) cycles of OPU as described. Following IVF, zygotes were cultured to the blastocyst stage whilst retaining individual donor identity.

### 3.1 Melatonin interacts with complex proteins during IVM to affect embryo development

Over the course of four OPU cycles, 420 GV oocytes were collected, matured and inseminated leading to the production of 254 blastocysts. The absence of CP in maturation media had no significant effect on the overall percentage of oocytes that cleaved following insemination by Day 2 of culture, although there was an indication (p=0.059) that the presence of melatonin during IVM reduced the percentage cleaved (**Table 1**). However, of oocytes cleaved, a greater (p < 0.001) percentage of those matured in the presence than absence of CP had progressed beyond the 6-cell stage (57.5 ± 6.72 vs 27.0 ± 5.36). There also appeared (p = 0.092) to be an interaction between CP and melatonin on the developmental progress of cleaved embryos, whereby adding melatonin in the presence of CP increased the percentage of embryos that developed beyond the 4-cell stage, whilst adding melatonin in the absence of CP decreased this percentage (**Table 1**).

**Table 1:** Effect of the presence or absence of complex proteins (CP), with or without 100 nM added melatonin, during *in vitro* oocyte maturation on subsequent embryo development to Day 8 following *in vitro* fertilization.

Complex proteins (CP)		Present		Absent		*p*-Value		
Melatonin (M), nM		0	100	0	100	CP	M	CPxM
Donor cycles, n		8	8	8	8			
A. Day 2 cleavage stage embryos								
Inseminated per donor, n		10.4 ± 1.16	13.6 ± 1.32	13.0 ± 1.28	13.6 ± 1.36	–	–	–
Cleaved of inseminated, %		97.5 ± 2.11	87.5 ± 5.67	94.0 ± 3.37	81.7 ± 6.62	–	0.059	–
3-4 cells of cleaved, %		15.5 ± 6.04	9.1 ± 4.75	28.0 ± 6.50	30.0 ± 7.30	0.011	–	–
5-6 cells of cleaved, %		21.9 ± 7.20	22.6 ± 5.74	39.0 ± 6.73	30.8 ± 6.57	0.052	–	–
>4 cells of cleaved, %		80.2 ± 6.04	90.0 ± 4.33	66.4 ± 5.90	64.2 ± 6.89	0.004	–	0.092
>6 cells of cleaved, %		48.5 ± 10.80	65.1 ± 8.04	28.4 ± 7.17	25.8 ± 7.88	<0.001	–	–
B. Day 6 embryos (≥ 12 -cell stage)								
Embryos of inseminated, %		80.1 ± 6.71	79.3 ± 5.90	56.5 ± 2.91	71.2 ± 6.76	0.032	–	–
Embryos of cleaved, %		82.6 ± 5.44	87.8 ± 4.26	61.2 ± 6.07	83.9 ± 4.83	0.028	0.002	–
Blastocysts of Day 6, %		14.7 ± 7.98	31.5 ± 8.26	22.3 ± 7.39	6.7 ± 4.99	–	–	0.028
C. Day 8 blastocysts								
Blastocysts of inseminated, %		61.4 ± 6.68	67.5 ± 5.48	52.3 ± 6.00	53.9 ± 6.28	0.088	–	–
Blastocysts of cleaved, %		62.9 ± 6.95	74.9 ± 5.50	56.8 ± 6.30	63.9 ± 6.77	–	–	–
Blastocysts of Day 6, %		78.5 ± 7.43	84.1 ± 6.22	92.7 ± 5.34	75.9 ± 8.20	–	–	0.035
Stage^‡^ 8 & 9 of Day 6, %		31.9 ± 6.90	45.7 ± 6.13	19.8 ± 4.93	17.3 ± 5.92	0.001	–	–
Stage^‡^ 8 & 9 of Day 8, %		40.7 ± 7.78	55.1 ± 6.43	21.7 ± 5.16	24.2 ± 5.51	<0.001	–	–
Stage^‡^ 7 (1), 8 & 9 of Day 8, %		71.8 ± 8.66	78.8 ± 6.63	53.9 ± 9.04	59.4 ± 9.54	0.019	–	–
Stage^‡^ 9 of Day 8, %		19.5 ± 4.80	34.6 ± 4.47	13.4 ± 2.68	6.6 ± 3.28	<0.001	–	0.019

^‡^Blastocyst Stage according to IETS (63): Stages 8 and 9 = hatching and hatched blastocysts respectively; Stage 7 = fully expanded. Stage 7(1) refers to morphological grade 1 (63) fully expanded Day 7 blastocysts.

Similar trends were observed at Day 6, with a greater (p = 0.032) percentage of inseminated oocytes progressing to and beyond the 12-cell stage in the presence than absence of CP. An interaction (p = 0.028) between CP and melatonin indicated that, whereas the inclusion of melatonin in IVM media containing CP increased the percentage of Day 6 blastocysts, its inclusion to defined IVM media (i.e., in the absence of CPs) decreased blastocyst yields (**Table 1**). This interaction was also evident by Day 8, for both Day 8 blastocysts as a percentage of Day 6 embryos (P=0.035) and hatched (i.e., IETS Stage 9) blastocysts of Day 8 blastocysts (p = 0.019). In general, however, the percentage of embryos that developed to advanced stages was greater in the presence than absence of CPs (**Table 1**).

### 3.2 Removal of CP during IVM alters transcripts involved in lipid and steroid metabolism in cumulus cells

**Figure 1:**
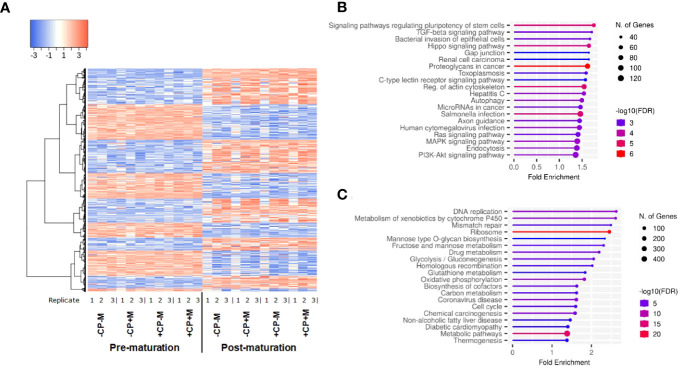
**(A)** Heatmap depicting cumulus-cell gene expression before and after *in vitro* maturation; **(B)** Top 20 KEGG pathways enriched for genes up regulated following maturation; **(C)** Top 20 KEGG pathways enriched for genes down regulated following maturation gene. Figures **(B, C)** generated by ShinyGO v0.76.3 (64).

Transcript expression was determined by 3’ mRNA-sequencing using RNA extracted from cumulus cells (CC) trimmed from Grade 1 COCs immediately before and following IVM. Cumulus cell transcript expression changed substantially following IVM. Between pre- and post-maturation 8,777 genes were differentially expressed, with 4,760 upregulated and 4,017 downregulated post-maturation (**Figure 1A**). Gene enrichment analysis of all differentially expressed transcripts post maturation highlighted upregulated transcripts within KEGG pathways associated with cell signaling in general, and downregulated transcripts within KEGG pathways associated with various aspects of cellular metabolism (Top 20 KEGG pathways represented; **Figures 1B, C**). When gene enrichment analysis was limited to transcripts upregulated >4-fold post maturation, more than 70 KEGG pathways were identified (**Supplementary Figure 1A**). These related mostly to processes such as inflammation, cytokine/immune response, cell proliferation, extracellular matrix (ECM), and intra-cellular signaling. In comparison, only seven KEGG pathways were identified for genes downregulated by >4-fold (**Supplementary Figure 1B**). These include genes involved in meiosis, nitrogen and glutathione metabolism, and cell cycle regulation.

**Figure 2:**
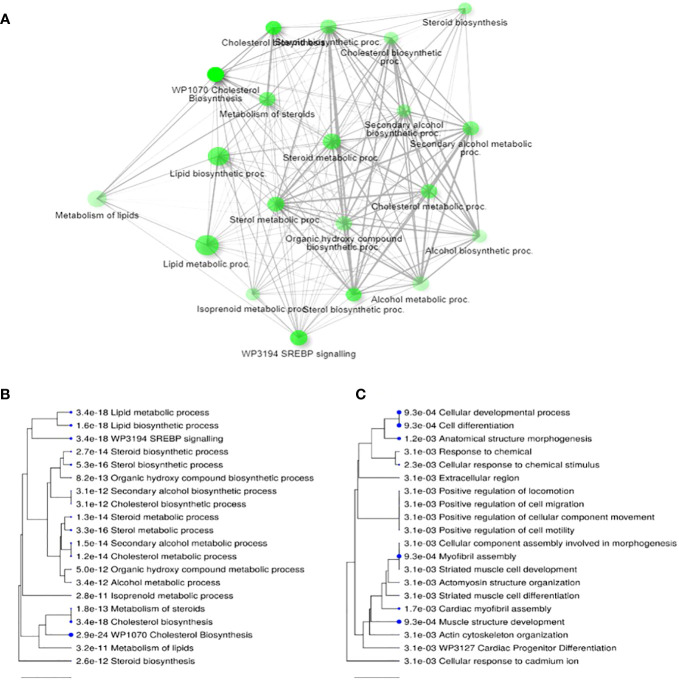
Differential transcript expression in cumulus cells following 24 h maturation in the presence or absence of complex proteins (CPs). **(A)** Network for top 20 enriched pathways for transcripts differentially expressed (i.e., up and down regulated) when CP are removed from IVM media; **(B)** Hierarchical clustering of top 20 pathways for upregulated transcripts in CP-free media; **(C)** Hierarchical clustering of top 20 terms for downregulated transcripts in CP free media. Note, in **(A)** two pathways are connected if they share 20% or more genes. Darker nodes are more significantly enriched; larger nodes contain more transcripts; thicker lines represent more overlapped transcripts. Concerning **(B)** and **(C)**, pathways with many shared transcripts are clustered together. Larger dots indicate greater statistical significance. Figures generated by ShinyGO v0.76.3 (64).

The removal of CP from maturation media resulted in differential expression of 77 genes (40 up and 37 down regulated) in post-maturational CC. Gene enrichment analysis indicated that removal of CP led to an upregulation of 27 genes related to lipid, cholesterol and steroid metabolism or biosynthesis processes (**Figures 2A, B**). Downregulated genes (**Figure 2C**) were predominantly involved in cellular organization including proliferation, motility, and apoptosis. Many of these genes are responsive to factors expected to be increased in the presence of CP, such as fatty acids (FA), ROS, cytokines, and growth factors (**Supplementary Table 1**).

Changes in post-maturational CC transcript expression were less evident when melatonin was added to IVM media. When melatonin was added in the presence of CP, only 22 genes were differentially expressed (3 up and 19 down regulated). Gene enrichment analysis indicated that many of these genes are involved in immune responses or are cytokine inducible (**Supplementary Table 2**). Of these 22 genes, 16 (1 up, 15 down regulated) are linked to metabolic functions, pre-dominantly glycolysis (**Supplementary Table 3**). There was no significant differential expression of transcripts when melatonin was added in the absence of CPs (i.e., -CP+M vs -CP-M). However, relative to the control group (+CP-M), 131 transcripts were differentially expressed when CP was removed and melatonin added (-CP+M). Also, a similar number of differentially expressed transcripts (81) were observed when CPs were removed in the presence of melatonin (-CP+M vs +CP+M). Many of the same genes/gene ontology terms were observed as for the removal of CPs, suggesting a dominant influence of CPs. However, a number of additional gene enrichment terms relating to downregulated genes for -CP+M vs +CP-M or +CP+M were observed. These included hippo, NF-κB and oxytocin signaling, regulation of insulin secretion, and responses to hypoxia (**Supplementary Figure 2**).

### 3.3 Removal of CP during IVM alters metabolism and mtDNA copy number in Day 8 blastocysts

**Figure 3:**
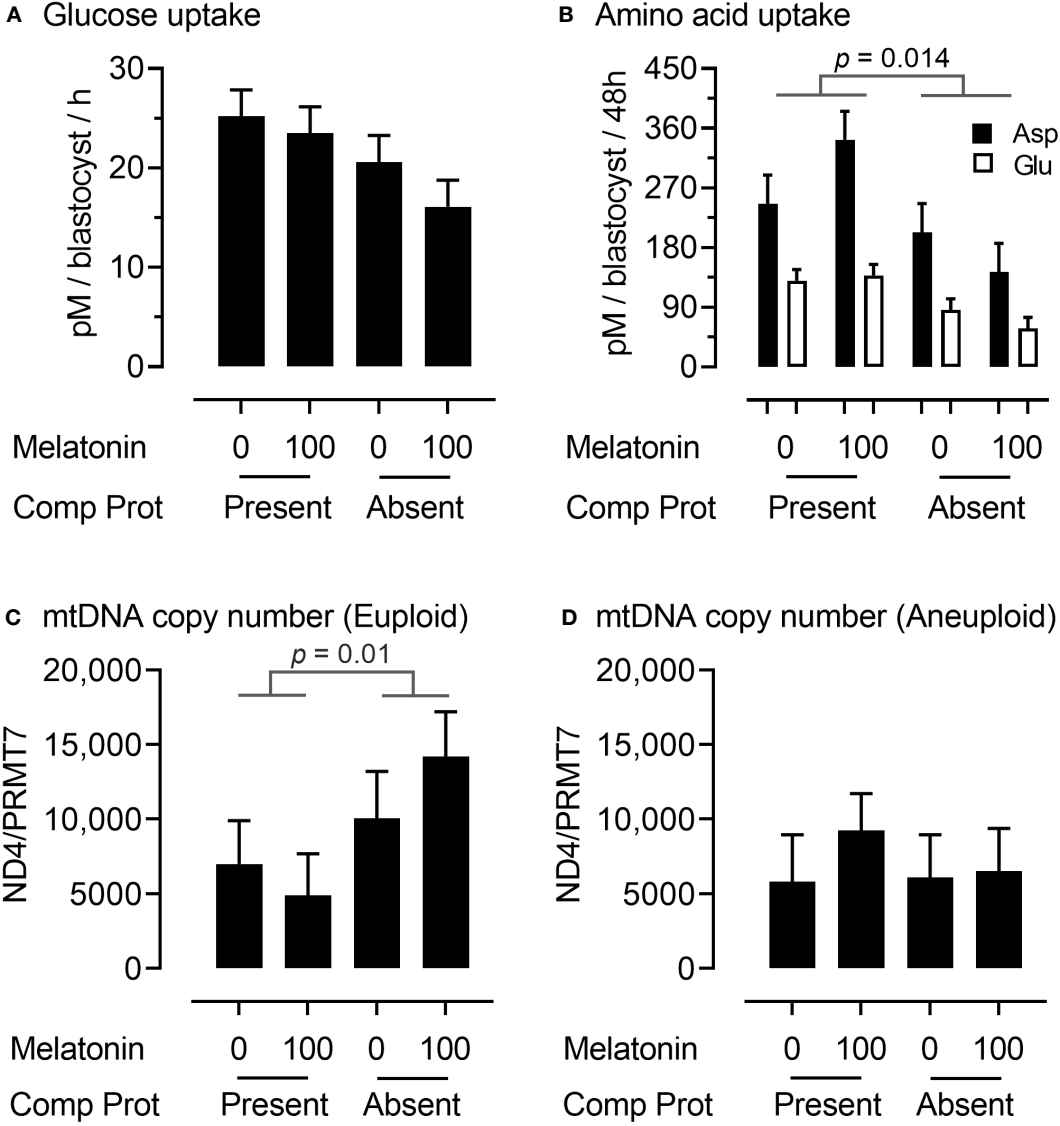
Embryo metabolism and embryo mitochondrial DNA copy number for oocytes matured in the presence or absence of complex proteins with or without the inclusion of 100 nM melatonin. Glucose **(A)** and aspartate and glutamate **(B)** uptake from media between Days 6 to 8 of embryo culture; mitochondrial DNA copy number (ratio of NADH dehydrogenase subunit 4 (*ND4*): Protein arginine N-methyltransferase 7 (*PRMT7)*) in euploid **(C)** and aneuploid **(D)** Day 8 blastocysts.

We next sought to characterize, non-invasively, glucose and amino acid metabolism of transitioning blastocysts between Days 6 to 8 of development from the analysis of spent culture media. Glucose uptake by embryos during this period did not differ significantly between treatments, although there was an indication that it declined when CP were removed from IVM media (**Figure 3A**). However, there was a decrease (p = 0.014) in the uptake of two amino acids (aspartate and glutamate) by embryos between Days 6 to 8 when CP were removed from IVM media (**Figure 3B**). The triglyceride content of Day 8 blastocysts was also determined. However, this did not differ significantly between treatment groups (data not presented).

To gain an insight into mitochondrial responses during these latter stages of embryo development, DNA was extracted from trophectoderm (TE) cells and mitochondrial DNA (mtDNA) copy number determined by qPCR using primers for the NADH dehydrogenase subunit 4 (ND4) normalized to a single copy-number gene (Protein arginine N-methyltransferase 7 (PRMT7). Interestingly, mitochondrial DNA copy number in TE cells was higher (p = 0.01) in euploid (**Figure 3C**) but not aneuploid (**Figure 3D**) Day 8 blastocysts derived from oocytes matured in the absence rather than the presence of CP.

### 3.4 No evidence that removal of CP affects the incidence of aneuploidy in Day 8 blastocysts

Chromosomal errors were assessed in immuno-dissected TE cells from Day 8 blastocysts by Karyomapping and Gabriel-Griffin plots (which indicate meiotic aneuploidy) and Signal Intensity data (BAF/LRR) which indicate overall aneuploidy (55, 58). Of the 123 TE (across all four treatments) tested, 25 (20.3%) were aneuploid. From these 25 TE, 30 errors were identified, of which whole chromosome errors (trisomy 40.0% [12/30], and monosomy 16.7% [5/30]) were the most common, followed by triploidy/hypotriploidy (26.7% [8/30]) (**Table 2A**). Neither the type of error identified, nor the incidence of aneuploidy, differed significantly between treatments. However, numerically, the lowest incidence of aneuploidy recorded (15.7%) was for oocytes matured in the presence of CP but without melatonin (i.e., +CP-M). The percentage aneuploidy for the other treatments were: +CP+M (22.2%), -CP-M (23.1%), and -CP+M (20.7%).

**Table 2:** Nature and incidence of chromosomal errors in Day 8 blastocysts.

	Overall	Parental origin		
		Dam	Sire	Embryo
A. Aneuploidy class				
Triploidy and hypotriploidy	8	1	4	3
Whole chromosome	20	16	1	3
Segmental	2	–	2	–
Total errors	30	17	7	6
B. Whole chromosome errors				
Trisomy	12	11	–	1
Mi	(10)	(10)	–	–
Mii	(1)	(1)	–	–
Mitotic	(1)	–	–	(1)
Tetrasomy	1	1	–	–
Chromosome Y disomy	1	–	1	–
Monosomy	5	4	–	1
Uniparental disomy (UPD)	1	–	–	1

Analyses undertaken on immuno-dissected trophectoderm cells.

The parental origin of aneuploidy was identified in all but six cases (**Table 2**). It was also possible to identify the developmental origin of 18 from 30 errors (in 25 affected TE samples). Of the 12 trisomies (**Table 2B**), 10 originated during meiosis I, one during meiosis II and one during mitosis. In addition, for 6 triploidies/hypotriploidies, two originated during MI, one during MII and three during mitosis. Finally, the single case of tetrasomy originated during MI. Although most errors occurred during meiosis I (66.7%), errors for the +CP-M group were only identified during either meiosis II or during mitosis.

### 3.5 Composition of IVM media generates unique patterns of DNA methylation in Day 8 blastocysts

To gain an insight into the epigenetic consequences of removing CP from IVM media, the inner-cell mass (ICM) of Day 8 blastocysts was isolated and analyzed for differential DNA methylation (56) between IVM treatment groups. Analyses revealed differences in CpG methylation for each of the six comparisons between the four IVM treatment groups (**Table 3**). Interestingly, the number of CpGs that were differentially methylated was similar in magnitude for each comparison, with a similar percentage that either gained or lost methylation.

**Table 3:** Differentially methylated (>10%) cytosine-phosphate-guanine dinucleotide (CpG) counts between the inner-cell mass (ICM) of Day 8 blastocysts derived from oocytes matured in the presence (+CP) or absence (-CP) of complex proteins (CP) with or without the inclusion of melatonin (0 v 100 nM).

General comparison	Specific comparison	CpG count	↑ Methylation	↓ Methylation
Standard IVM (+CP-M) vs other combinations	+CP-M vs +CP+M	2878	1408 (48.9%)	1470 (51.15)
	+CP-M vs -CP-M	2698	1293 (47.9%)	1405 (52.1%)
	+CP-M vs -CP+M	2105	1213 (57.4%)	892 (42.6%)
Standard IVM + Melatonin vs Defined	+CP+M vs -CP-M	3382	1610 (47.6%)	1772 (52.4%)
	+CP+M vs -CP+M	2576	1478 (57.4%)	1098 (42.6%)
Defined IVM vs Defined + Melatonin	-CP-M vs -CP+M	2290	1331 (58.1%)	959 (41.9%)

Arrows indicate gain (↑) or loss (↓) of methylation (percentage altered in parentheses).

**Figure 4:**
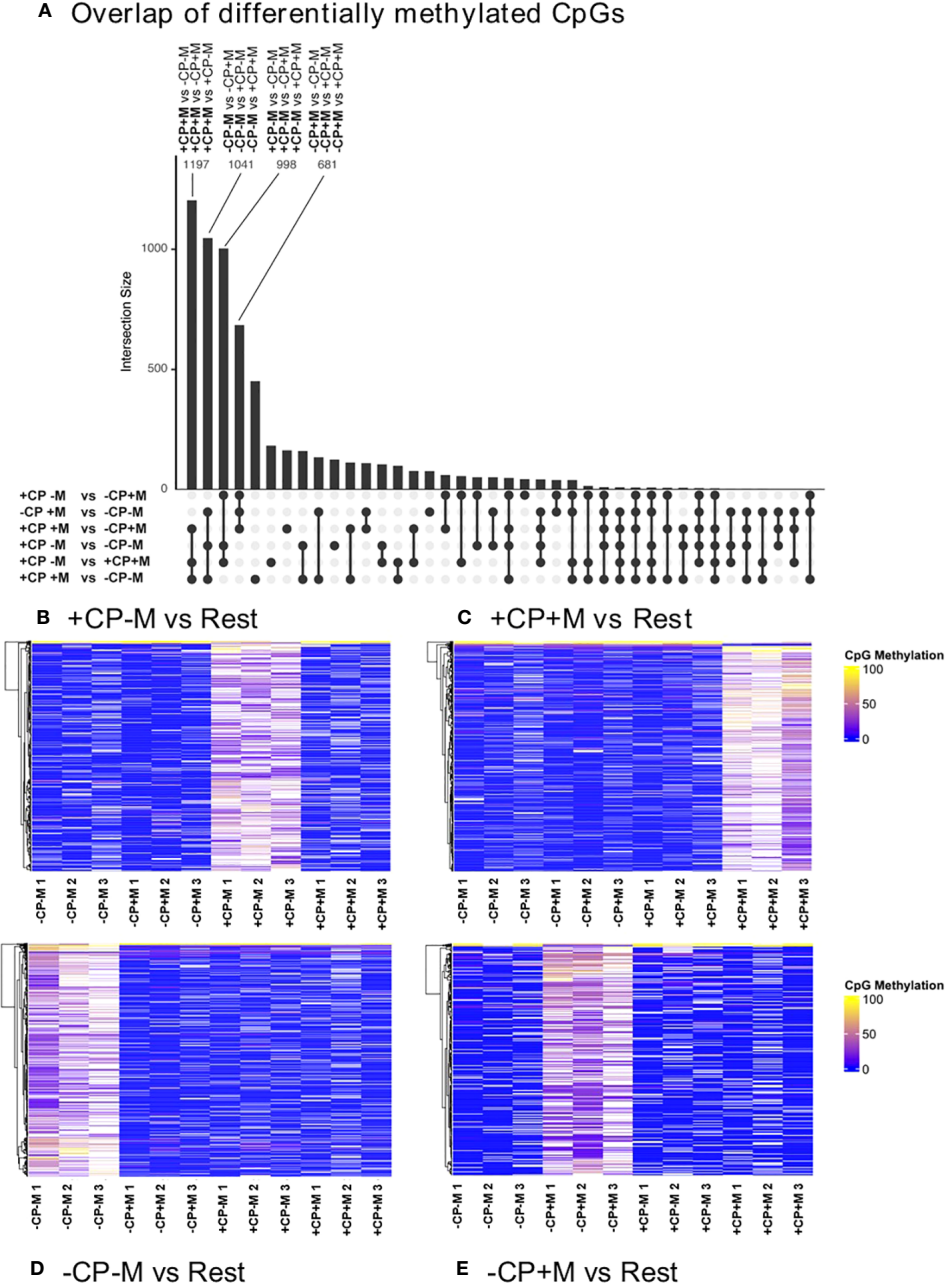
Overlapping differentially methylated (>10%) CpGs (ranked by number) identifies four pairwise comparisons between each of the four treatment groups with each of the other three treatments **(A)**; and heat maps for each of these pairwise comparisons which, in each case, reveal that the majority of CpG methylation was high in the primary treatment group and low in the other three (**B–E**). These heat maps present the three biological replicates for each treatment. Analyses were restricted to DNA extracted from the inner-cell mass of pools of five fully expanded blastocysts derived from oocytes matured in the presence (+CP) or absence (-CP) of complex proteins (CP) with (+M) or without (-M) the inclusion of melatonin (0 v 100 nM).

Further analysis sought to identify overlapping differentially methylated CpGs between each of the six comparisons for the four IVM treatment groups. This analysis revealed a high number of differentially methylated CpGs for four pairwise comparisons where, in each case, CpG methylation was greater in the primary treatment group compared to the other three (**Figure 4A**). When presented as a heat map this analysis visually identified sets of relatively ‘hypermethylated’ CpGs unique to each of the four IVM treatment groups (**Figures 4B–E**).

Genes identified within 1000 bases of these uniquely hypermethylated CpGs were next submitted to gene enrichment analysis (GeneTrail 3.2 – https://genetrail.bioinf.uni-sb.de/start.html). ‘Metabolic process’ accounted for approximately one quarter of genes, regardless of treatment, with 23.8%, 25.1%, 28.4% and 23.5% of genes (+CP-M, +CP+M, -CP-M and -CP+M respectively) relating to this term. From these genes, processes unique to the individual treatments were further examined. When CP were removed (i.e. -CP-M), there was enrichment for the terms ‘Catabolic process’ and ‘Lipid metabolic process’, for which many of the genes overlapped, suggesting an influence on lipid catabolism. Terms enriched for the treatment group where both CP and M were present (i.e., +CP+M) included ‘Negative regulation of biosynthetic process’ (from which none of the genes overlapped with the other metabolic term ‘Lipid biosynthesis’). For ‘Lipid biosynthesis’ genes predominantly related to cholesterol, sterol and long-chain fatty acid biosynthesis. Lipid related genes were also identified when CP was removed and M added (i.e., -CP+M treatment group), with enrichment for the term ‘Response to lipid’.”

## 4 Discussion

A number of important findings emerge from the current study. Importantly, we report that, at least in the context of GV-intact oocytes originating from stimulated (‘coasted’) cycles of OPU, modest yields of transferrable-quality blastocysts can be generated following the complete removal of CP from IVM media. Also, whilst the addition of melatonin to IVM media enhances embryo development in the presence of CP [thereby representing an improvement to standard practice (54, 55, 60)], it impairs embryo development when added in the absence of CP. Interestingly, these interactive effects of CP and melatonin on embryo development do not appear to be mediated by either the nature or incidence of chromosomal errors arising during IVM, as statistically these were unaffected by treatment (**Table 2**). A more rigorous assessment would require a larger sample size. However, the lack of numerical differences between treatments indicates that any effects, should they exist, are likely to be small. Instead, they most likely arose due to metabolic perturbations occurring within the cumulus-oocyte complex (COC), together with impaired cell-to-cell communication, reduced lipid availability and utilization. At present, this supposition is based primarily on altered global transcript expression in cumulus cells, which indicates that removal of CP from IVM media leads to an increase in molecular pathways associated with sterol (particularly cholesterol) biosynthesis, and a reduction in pathways associated with cell structure, organization and signaling (**Figure 2**). Anecdotally, it was also noted at the time that the expanded cumulus-oophorus was ‘less coherent’ (i.e., there was a loose association between cumulus cells and the oocyte) following IVM in the absence of CP. In contrast, the inclusion of melatonin to CP supplemented IVM media led to a reduction in expression of transcripts linked to glucose metabolism, primarily glycolysis. Ultimately, the composition of IVM media led to distinct metabolic and epigenetic signatures in fully expanded and hatching/hatched blastocysts, linked to mitochondrial biogenesis and associated glucose, fatty acid and cholesterol metabolism. Collectively, these findings highlight the importance of lipid metabolism (in this instance derived from CP) by the COC during IVM.

### 4.1 Developmental and metabolic impact of removing complex proteins during IVM

Whilst post-fertilization development to the blastocyst stage was impaired following removal of CP from IVM media in the current study, reasonable yields of Day 8 blastocysts (~53% of presumptive zygotes) were nevertheless obtained. This observation is generally consistent with previous reports on the effects of removing CP (which can come in the form of serum and/or albumin) from IVM media (81–85). These studies, however, differ from the current in that they invariably utilized GV-intact oocytes retrieved from non-stimulated abattoir derived ovaries, whereas we recovered GV-intact oocytes from FSH-stimulated (‘coasted’) cycles of OPU. Oocytes originating from such cycles are developmentally more competent (54, 55, 86) having partially undergone cytoplasmic and molecular maturation *in vivo* prior to aspiration (87, 88). Further variability in responses to CP removal between studies can be attributed to differences in basal maturation medium, origin and inclusion level of albumin and/or serum, nature and inclusion level of alternative macromolecules (e.g., polyvinyl alcohol vs PVP), and presence or absence of different combinations of hormones (e.g., FSH) and growth factors (e.g., EGF).

Complex proteins in the form of serum and albumin comprise numerous but poorly defined components (89) that can aid oocyte maturation (85). Removal of these components, as undertaken in the current study, can therefore provide important insights into their collective function. Here, for example, we report that the absence of CP during IVM leads to an increase in mRNA expression by cumulus cells for processes linked to lipid and sterol metabolism (**Figures 2A, B**), and a decrease in mRNA expression linked to cellular organization (**Figure 2C**). Further analyses (ShinyGo v0.76.3) of upregulated transcripts listed in **Supplementary Table 1** identified 7 out of 20 (with a >200-fold enrichment) within the KEGG ‘steroid biosynthesis’ pathway. Specifically, these 7 transcripts (*FDFT1, SQLE, LSS, CYP51A1, TM7SF2, MSMO1* and *NSDHL*) occupy 10 consecutive enzymatic steps in the biosynthesis of cholesterol from farnesyl pyrophosphate via 14-demethyl-14-dehydrolanosterol (i.e., follicular fluid meiosis-activating sterol (FF-MAS)); an intermediate in cholesterol biosynthesis that serves to promote meiotic resumption under the influence of FSH (90). Also upregulated in this pathway was *DHCR7*, which catalyzes the final step of cholesterol biosynthesis. Other notable upregulated transcripts linked to cholesterol uptake and metabolism listed in **Supplementary Table 1** include *LDLR* (endocytosis of cholesterol by the cell), *ACLY* (conversion of citrate to actetyl-CoA, from which cholesterol is derived via farnesyl pyrophosphate), *ACAT2* (esterification of cholesterol), *HMGCR* (a rate limiting enzyme for cholesterol and isoprenoid synthesis), *HMGCS1* (early step in cholesterol biosynthesis involving the condensation of acetyl-CoA with acetoacetyl-CoA), and *INSIG1* (involved in SREBP-mediated regulation of cholesterol biosynthesis). Complex proteins therefore serve as a key source of cholesterol for the COC during IVM, removal of which leads to compensatory upregulation of sterol biosynthetic pathways by cumulus cells. Indeed, the absence of serum during IVM is known to reduce intracellular levels of non-polar lipids (including cholesterol) in oocytes (91, 92).

At present, the timing of these metabolic responses during IVM is not defined but, given the important role of oocyte-derived factors such as GDF9 and BMP15 in regulating cholesterol biosynthesis in cumulus cells (93), is likely to coincide with the period leading up to GV-breakdown and expansion of the cumulus oophorus, both of which are initiated between 6-9 h after follicular aspiration (94–96). Indeed, a significant amount of trafficking of small molecules and transcripts between cumulus cells and the oocyte occur via intact transzonal projections during this period (97). Nonetheless, metabolic processes and exchange of molecules between cumulus cells and the oocyte, that promote post-fertilization development, continue beyond this stage of maturation (98). Data from the current study, however, can shed no further light on these temporal aspects as the observed decrease in transcripts linked to metabolic processes, and increase in transcripts linked to cell signaling, inflammation and formation of the ECM, occurred over 24 h of IVM (**Figure 1**).

The decline in embryo development following removal of CP during IVM (**Table 1**) was matched by metabolic alterations in blastocysts between Days 6 and 8 of culture (**Figure 3**). Consistent with these findings are reports in the mouse that both glucose and aspartate consumption are reduced in ‘slower’ developing blastocysts, together with transcripts for two glucose transporters (*Slc2a1, Slc3a3*) and glutamic-oxaloacetic transaminase (*Got1*) (99). This enzyme is centrally involved in the malate-aspartate shuttle which, in turn, regulates tricarboxylic acid cycle activity within the mitochondrion (100). The increase in mtDNA copy number in chromosomally normal blastocysts (**Figure 3C**) further points to a potentially ‘programmed’ dysregulation of energy metabolism in embryos derived from oocytes matured in the absence of CP.

### 4.2 Melatonin and complex proteins interact during IVM to impact embryo development

In contrast to the effects of removing CP, the impact of adding melatonin to IVM media was more subtle, being largely dependent on the presence of CP, and was most apparent at later stages of embryo development (**Table 1**; **Figure 4**), although there was an indication that the percentage cleaved following insemination was reduced. The motivation for adding melatonin to our standard IVM media, which contains CP, stemmed from its known actions as a metabolic regulator (44) and antioxidant (38). Melatonin is a natural component of follicular fluid, being derived from both systemic and local sources (51, 101), and its inclusion during IVM in media comparable to our +CP standard has been found to enhance oocyte maturation and embryo development (38, 42, 46).

Reduced rates of cleavage (102) and reduced polyspermy (103) can occur following the addition of melatonin during IVM. This may be due to increased/improved cortical granule distribution which has been observed for oocytes matured in the presence of melatonin (104, 105). Interestingly, incubating sperm with melatonin also reduces its ability to bind to oocytes (106) and leads to lower polyspermy (107), suggesting melatonin may be an important factor in regulating fertilization. The implication is that the improved percentage of Day 6 blastocysts of those that had cleaved following melatonin treatment (**Table 1B**) may have been a consequence of a reduced number of polyspermic embryos on Day 2, although this remains to be verified.

In the current study, the addition of melatonin to our standard media (i.e., +CP+M vs +CP-M) altered the expression of 22 transcripts in cumulus cells (**Supplementary Tables 2, 3**). These were mostly linked to immune function and metabolism (particularly glycolysis), and the addition of melatonin downregulated the expression of 20 of these transcripts. Pathway-fold enrichment for six transcripts (i.e., *KRT8, KRT18, DSG, PDGFRA*, *AKAP12* and *CSRP3*) stood apart from the rest (**Supplementary Table 2**). The first five of these six transcripts are known to be expressed within the ovarian follicle and are linked to cell proliferation and apoptosis (108–111). Their downregulation, therefore, could tentatively be associated with more ‘viable’ and ‘mature’ COCs.

There was no significant effect of melatonin on cumulus-cell transcript expression when it was added to IVM media in the absence of CP (i.e., -CP+M vs -CP-M). In contrast, the expression of 118 transcripts differed between the two treatments that led to the greatest difference in embryo development (i.e., -CP+M vs +CP+M; **Table 1**). However, as reported earlier, the increase in transcript expression observed following the removal of CP in the presence of melatonin was related mostly to sterol and lipid metabolism. Of the 68 downregulated transcripts, eight overlapped within and between identified KEGG pathways and GO Biological Processes (**Supplementary Figures 2C, D**). In cumulus cells, these transcripts are linked to cAMP and calcium signaling [*PRKACB, RYR2* (112, 113)], mitochondrial metabolism [*PDK3, UCP2* (114, 115)], apoptosis [*MT1E, FAM162A (* 116 *)*], and prostaglandin synthesis [*PLA2G4A, PTGS2* (117, 118)]. Additionally, four of these transcripts (*UCP2, FAM162A, PTGS2, PDK3*) were identified as hypoxia inducible. These have been implicated in preferential glycolysis in tumor cells (119–122). The addition of melatonin has been shown to inhibit such preferential glycolysis (123), and to reduce proliferation in cancer (124) and granulosa cells (125) *in vitro*. Interestingly, it has also been shown that this inhibition is less effective (126) or indeed reversed (53) in the presence CP. Whilst these processes are fundamental to the successful maturation of COCs, the link to observed differences in embryo development (**Table 1**) is tenuous at present given their disparate nature and the limited number of underpinning transcripts.

### 4.3 The ploidy status of blastocysts is unaffected by the composition of IVM media

Chromosomal abnormalities are a major cause of early embryo loss and pregnancy failure in both humans and cattle (58, 127, 128), and there are concerns that procedures involved in ART could exacerbate their incidence (129). However, ovarian stimulation in cattle, at least using the ‘coasting’ protocol described herein, is not responsible *per se* for inducing aneuploidy (55). The implication is that, because most errors reported are of meiotic (mostly MI; **Table 2**) origin (55, 58), and the incidence of aneuploidy is greater for IVP than for *in vivo* derived embryos (55), a proportion of these errors arise a consequence of ‘stresses’ associated with oocyte recovery (OPU) and/or IVM.

In the current study, 20% of blastocysts harbored some form of chromosomal abnormality; a level comparable to that (14% to 24%) of our previous studies (55, 58). Yet, neither the removal of CP nor the addition of melatonin to IVM media appeared to affect the incidence of aneuploidy; although it could be argued that experimental power wasn’t great enough to provide a robust statistical analysis for this outcome across the four treatment groups. Rather, the increased incidence of aneuploidy associated with these procedures is more likely a consequence of the precocious onset of meiotic resumption in the oocyte upon aspiration from the ovarian follicle (130), combined with fluctuations in the micro-environment (i.e., mechanical stresses, osmolarity, temperature and pH) during processing (129).

### 4.4 Composition of IVM media determines unique DNA methylation ‘signatures’ in blastocysts

The striking feature to emerge from the analysis of DNA methylation within the ICM of Day 8 blastocysts is the existence of distinct subsets of methylated CpGs specific to each of the four IVM treatment groups (**Figure 4**). This indicates that modest alterations to the composition of IVM media for GV oocytes transiting to MII prior to fertilization can lead to heritable epigenetic modifications to DNA methylation in fully expanded and hatching blastocysts. Although the experimental conditions and treatments were not similar, such observations are not unprecedented in that elevated concentrations of non-esterified fatty acids during IVM can lead to modified DNA methylation in both bovine and porcine embryos (131, 132).

Considering each of the four treatment combinations in the current study (**Figure 4**), together with the selected biological processes depicted in **Supplementary Table 4**, the data indicate that removal of CP and/or addition of melatonin during IVM increase CpG methylation in blastocysts for the genes listed which, in these examples, are associated with lipid metabolism. This observation implies that the associated biological processes portrayed in **Supplementary Table 4** are likely to fall under greater epigenetic regulation specific to each IVM treatment. Curiously, CpG methylation was also greater in regulatory regions of genes involved in lipid metabolism for blastocysts derived from heifers undergoing similar OPU procedures to those of the current study (133), suggesting that these metabolic pathways are more precisely regulated epigenetically at this stage of development.

Despite the presence of unique CpG profiles in the current study, there was a degree of overlap between treatments with respect to biological processes, particularly related to lipid metabolism and response to lipids more generally (**Supplementary Table 4**). Some notable genes associated with differential CpG methylation (**Supplementary Table 4**), with relatively high fold enrichment, were *COQ2* (biosynthesis of CoQ10 involved in the mitochondrial electron transport chain), *ACACA* (rate limiting enzyme in fatty acid synthesis), *HADHB* (β-oxidation of long-chain fatty acids within the mitochondrion), *LRP6* (LDL endocytosis and WNT signaling), and *FOXA1* (DNA-binding chromatin modifier with down-stream links to metabolism). At this juncture, due to the number and dispersal of differentially methylated CpGs, it would be problematic to associate these epigenetic differences in DNA methylation to transcript expression in blastocysts and to subsequent developmental outcomes; this would require more in-depth analyses. Also, the functional significance of the biological processes listed, and genes contained therein, await further investigation.

### 4.5 Reflections on cytogenetic and epigenetic analyses

A strength of the current study is that immunodissection of Day 8 blastocysts allowed us to undertake both cytogenetic and epigenetic analyses in the same cohort of embryos. We previously reported a high degree of concordance between the TE and ICM in both the nature and incidence of chromosomal errors (55), so that it was not necessary to analyze both lineages. However, there are recognized differences in chromatin organization and CpG methylation (56, 134–136), as well as in transcriptional profile (137) between these two lineages, so that lineage separation prior to DNA methylation analyses can be considered a refinement over whole blastocyst analyses which would otherwise confound these differences. As the ICM gives rise to the embryo proper it made sense to prioritize this lineage; an approach adopted by others (e.g., 138). However, greater culture-induced differences in regional CpG methylation were observed in TE than ICM cells in bovine embryos at around Day 7/8 (56) and Day 17 (139), so that IVM-induced epigenetic effects reported in the current study may be an underestimate of the true magnitude across both lineages. Furthermore, the pooling of ICMs required for DNA extraction prior to library preparation prohibited an assessment of the interactive effects of culture and embryo sex on CpG methylation, as reported by others (140). However, ICMs in the current study were pooled from stage-matched blastocysts and the percentage male embryos in our system is consistently around 58%; it appears to be insensitive to interventions such as those reported in the current study (55, 56).

## 5 Conclusions

In the current study, the absence of CP during IVM led to modest reductions in embryo development, whilst the effect of added melatonin was beneficial in the presence, but detrimental in the absence, of CP. Interactive effects of CP and melatonin on embryo development were not associated with chromosomal errors arising during IVM. Instead, they were mediated in part via modifications to metabolism, predominantly of lipids, by COCs during IVM. This, in turn, resulted in a related metabolic and epigenetic legacy detectable in fully expanded and hatching blastocysts several days later. These outcomes lay the foundation for future studies that seek to develop defined (protein free) systems for the *in vitro* maturation of mammalian oocytes. They highlight the importance of lipid, particularly sterol, metabolism during this period, and indicate that developmental and epigenetic consequences may persist beyond the point of embryo transfer.

## Data availability statement

The datasets presented in this study can be found in online repositories. The names of the repository/repositories and accession number(s) can be found in the article/**Supplementary Material**.

## Ethics statement

The animal studies were approved by AWERB, University of Nottingham, Nottingham, NG7 2RD, UK. The studies were conducted in accordance with the local legislation and institutional requirements. Written informed consent was obtained from the owners for the participation of their animals in this study.

## Author contributions

DT: Conceptualization, Formal Analysis, Investigation, Methodology, Writing – original draft, Writing – review & editing. GG-A: Investigation, Writing – review & editing. WK: Investigation, Methodology, Writing – review & editing. RS: Investigation, Methodology, Writing – review & editing. FS: Formal Analysis, Writing – review & editing. GS: Formal Analysis, Methodology, Writing – review & editing. CC-R: Formal Analysis, Writing – review & editing. AH: Methodology, Writing – review & editing. RL: Conceptualization, Methodology, Writing – review & editing. M-AS: Conceptualization, Writing – review & editing. RE: Formal Analysis, Methodology, Writing – review & editing. DG: Formal Analysis, Funding acquisition, Methodology, Project administration, Supervision, Writing – review & editing. KS: Conceptualization, Formal Analysis, Funding acquisition, Investigation, Methodology, Project administration, Resources, Supervision, Writing – original draft, Writing – review & editing.

## Appendix A

Supplementary data.
