# Supplementary material for: Developmental, cytogenetic and epigenetic consequences of removing complex proteins and adding melatonin during in vitro maturation of bovine oocytes
Source: Front Endocrinol (Lausanne). 2023 Oct 23;14:1280847. doi: 10.3389/fendo.2023.1280847 (PMC10647927; doi:10.3389/fendo.2023.1280847)
Supplement: Supplementary file 1 [file DataSheet_1.docx]

**Supplementary Material**

**
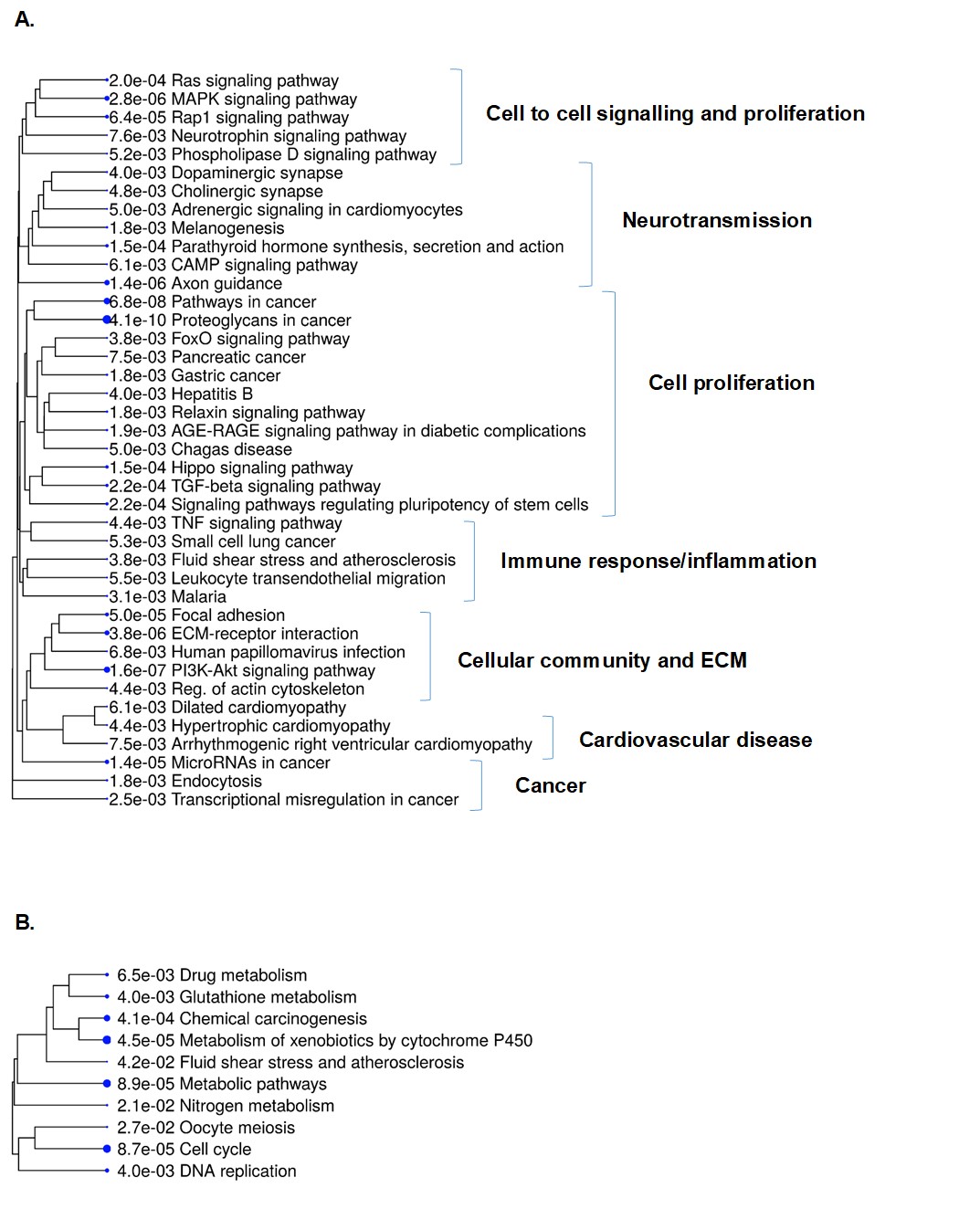
**

**Supplementary Figure 1.** KEGG pathways related to transcripts >4-fold differentially expressed between cumulus cells collected before and after *in vitro* maturation irrespective of treatment. Hierarchical clustering of the top 40 KEGG pathways related to transcripts up regulated after maturation (**A**) and all KEGG pathways related to transcripts downregulated after maturation (**B**). Figures generated by ShinyGO v0.76.3 (1)

**
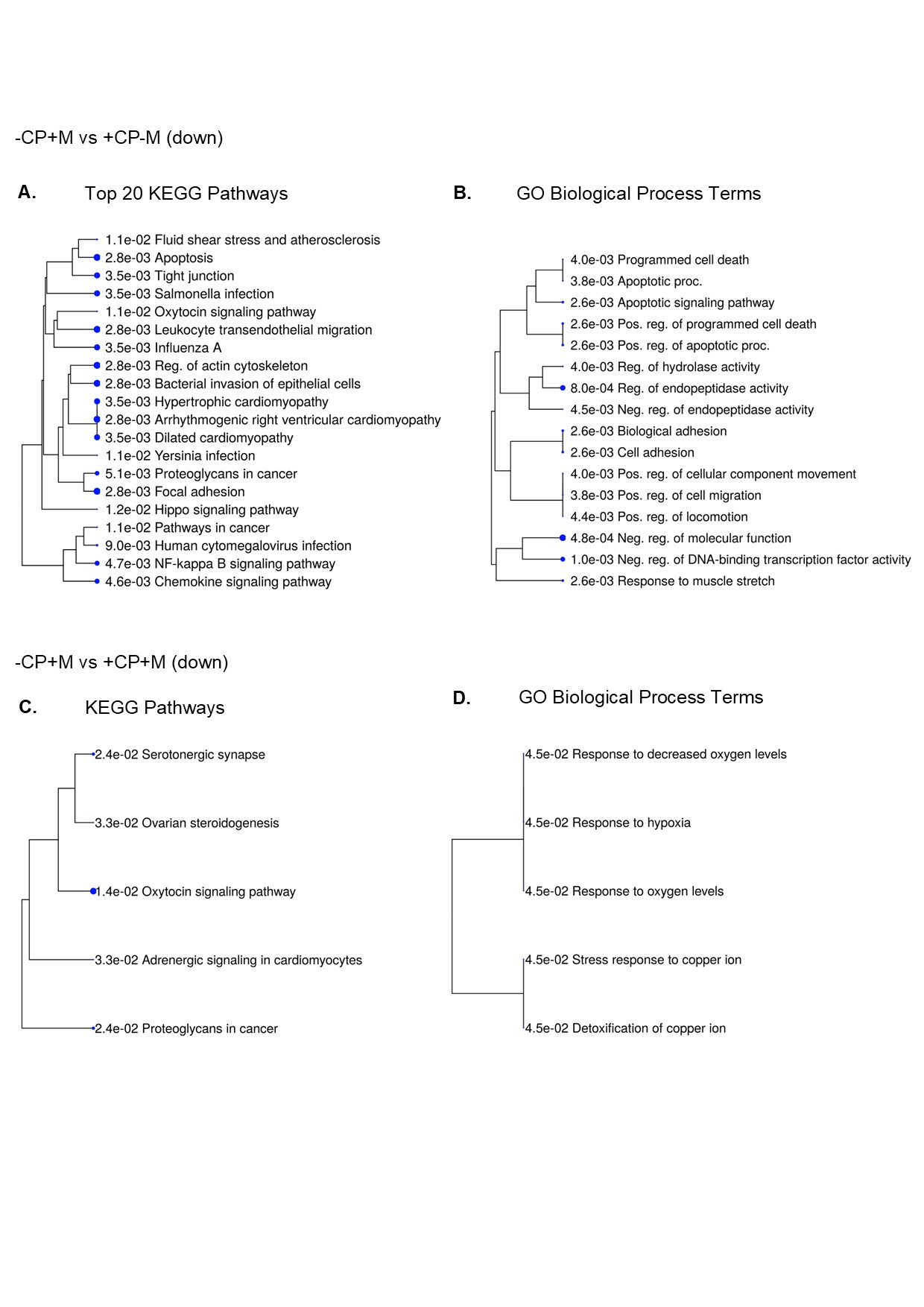
**

**Supplementary Figure 2**. Gene enrichment analysis for genes downregulated in cumulus cells treated with media containing melatonin but not complex proteins, as compared to cumulus cells treated with media containing complex proteins, without (A; B) or with (C; D) melatonin. Clustering occurs where pathways have genes in common. FDR is reported preceding the pathway name, and the size of the dot at the tip of the branch indicates the FDR relative to other pathways within the tree. Figures generated by ShinyGO v0.76.3 (1)

**Supplementary Table 1. Complex Proteins Removed** - Functional description (from GeneCards) for genes differentially expressed in cumulus cells after maturation in media with CP removed.

| **GeneName** | **Lipid/steroid related** | **Description** |
| --- | --- | --- |
|  | **Or** |  |
| **Upregulated** | **Function** |  |
| *SLCO2B1* | yes | Steroid transport |
| *RF00493* |  | Non-coding |
| *PRG4* |  | Codes for a glycoprotein and acts as a growth factor |
| *ACTA1* | cell growth/motility | Involved in cell motility, structure and integrity |
| *TM7SF2* | yes | Involved in cholesterol biosynthesis |
| *INSIG1* | yes | Cholesterol metabolism, lipogenesis, and glucose homeostasis |
| *SERPINA11* |  | Serine protease inhibitor |
| *HMGCS1* | yes | Ketogenesis and cholesterol biosynthesis |
| *CYP51A1* | yes | Synthesis of cholesterol, steroids and other lipids |
| *LPIN1* | yes | Triglyceride synthesis. |
| *IL4R* | apoptosis/cell proliferation | IL-4 receptor |
| *GPRC5A* |  | Induced by retinoic acid and modulates EGFR signaling |
| *MFSD2A* | yes | Lysophosphatidylcholine transporter |
| *DHCR7* | yes | Cholesterol biosynthesis |
| *IDI1* | yes | Cholesterol biosynthesis |
| *ENSBTAG00000047970* |  |  |
| *FADS2* | yes | Highly unsaturated fatty acid biosynthesis |
| *LIPE* | yes | Steroid hormone biosynthesis and triglyceride lipolysis |
| *FDFT1* | yes | Cholesterol biosynthesis |
| *SQLE* | yes | Sterol biosynthesis |
| *HMGCR* | yes | Cholesterol synthesis |
| *PALMD* |  | Regulation of cell shape |
| *SRGN* | ECM | Influences MMP2 and TNF-alpha secretion |
| *MVD* | yes | Cholesterol biosynthesis |
| *LDLR* | yes | Cholesterol metabolism and endocytosis |
| *LSS* | yes | Cholesterol, steroid hormones, and vitamin D synthesis |
| *NSDHL* | yes | Cholesterol biosynthesis and endocytosis |
| *MSMO1* | yes | Cholesterol biosynthesis |
| *FDPS* | yes | Isoprenoid biosynthesis |
| *ACAT2* | yes | Cholesterol biosynthesis |
| *RAMP1* |  |  |
| *PTPRN* |  | Insulin secretion (glucose responsive) |
| *FASN* | yes | Biosynthesis of long-chain saturated fatty acids |
| *PLCL2* | yes | Phosphatidylinositol-mediated signaling |
| *QPRT* |  | NAD synthesis and metabolism |
| *TMEM97* | yes | Cholesterol homeostasis |
| *B4GALT5* | yes | Synthesis of lactosylceramide |
| *FGF2* | yes | Mitogen/regulator of cell survival, division, differentiation, migration |
| *MAP7D2* | cell structure | Microtubule cytoskeleton organization |
| *ACLY* | yes | Regulates aerobic glycolysis and de novo lipid synthesis |
| ***Downregulated*** |  | |
| *RAB11A* | indirectly - cholesterol transport | Endocytosis and cholesterol transport |
| *PDK3* | response to fatty acid | Regulates aerobic respiration, glucose and fat metabolism. |
| *MT1E* | adipogenesis | Insulin regulation |
| *S100A10* |  | Exocytosis and endocytosis |
| *CSTB* |  | Thiol proteinase inhibitor |
| *LGALS1* | apoptosis/cell proliferation | Hypoxia induced, regulates cell cycle |
| *PDGFRA* | cell proliferation | Regulates cell proliferation, survival and chemotaxis |
| *PHLDA1* | apoptosis/cell proliferation | Regulates apoptosis, IGF1 stimulated |
| *TM4SF1* | cell growth/motility | Regulates cell development, activation, growth and motility |
| *NTS* | response to fatty acid | Fat metabolism |
| *ACTG1* | cell motility | Cytoskeleton and cell motility |
| *SEMA6D* | cell growth/motility | Neuronal development, cell proliferation and migration |
| *CTHRC1* | ECM | Modulator of Wnt signalling, collagen deposition |
| *GAL* |  | Supresses insulin, regulates energy homeostasis |
| *FLNB* | ECM | Connects cell membrane to cytoskeleton |
| *WASF1* |  | Regulates actin cytoskeleton |
| *LXN* |  | Cell proliferation, inflammation |
| *ID3* | response to fatty acid | Regulates cell differentiation, proliferation and motility |
| *TPD52L1* | apoptosis/cell proliferation | Cell proliferation and calcium signaling, indirectly apoptosis |
| *CSRP3* | cell growth | Gene regulation, cell growth and differentiation |
| *ANKRD1* |  | IL-1/TNF-alpha induced, regulates immune response/apoptosis |
| *SHTN1* | Cell movement | Neuron migration and polarization |
| *MSX1* | apoptosis/cell proliferation | Cell proliferation, craniofacial and limb development |
| *GSTM1* | ROS responsive/Antiox | Reduces ROS |
| *TNFSF18* |  | TNF family cytokine, modulates NF-kappa-B and STAT1 |
| *NCAM1* | apoptosis/cell proliferation | Neurogenesis, cell migration and ECM and signal transduction |
| *ID1* | apoptosis/cell proliferation | Cellular growth, senescence, differentiation, apoptosis |
| *S100A2* | ROS/TGFb/INFa responsive | Cell cycle progression/differentiation, proliferation, glycolysis |
| *CRYBG1* |  | Cytoskeletal regulation, carbohydrate binding |
| *PTGIS* | Prostaglandin | Prostanoid biosynthesis |
| *GNG2* | apoptosis/cell proliferation | Cell proliferation, migration, signalling and apoptosis |
| *CD109* | apoptosis/cell proliferation via TGFb | Regulates TGF-beta signaling, cell proliferation and apoptosis |
| *ACTC1* | apoptosis/cell proliferation | Cell motility, inhibits apoptosis |
| *DPYSL3* | cell movement | Actin filament/cell projection organization, cell migration |
| *MMP9* | ECM | ECM breakdown, cell migration |
| *CLDN1* | cell movement | Cell adhesion, hypoxia responsive |

([www.genecards.org](https://www.genecards.org/)) (2) (Stelzer et al. 2016)

**Supplementary Table 2. Melatonin Added** - Top 25 gene enrichment analysis terms for genes differentially expressed in cumulus cells when melatonin is added to maturation media (in the presence of complex proteins)

| **Pathway** | **Enrichment FDR** | **Fold enrichment** | **n genes** | **Pathway genes** | **Genes (up, down)** |
| --- | --- | --- | --- | --- | --- |
| **Keratin filament** | 0.0148 | 172.84 | 2 | 5 | *KRT8 KRT18* |
| **Hepatocyte apoptotic proc.** | 0.0148 | 144.03 | 2 | 6 | *KRT8 KRT18* |
| **Keratinization** | 0.0162 | 108.02 | 2 | 8 | *KRT18 DSG2* |
| **Formation of the cornified envelope** | 0.0162 | 108.02 | 2 | 8 | *KRT18 DSG2* |
| **Cardiac myofibril assembly** | 0.0162 | 108.02 | 2 | 8 | *PDGFRA CSRP3* |
| **Protein kinase c signaling** | 0.0162 | 108.02 | 2 | 8 | *CSRP3 AKAP12* |
| **Cardiac muscle cell development** | 0.0324 | 50.83 | 2 | 17 | *PDGFRA CSRP3* |
| **Scaffold protein binding** | 0.0375 | 45.48 | 2 | 19 | *KRT8 KRT18* |
| **Myofibril assembly** | 0.0486 | 39.28 | 2 | 22 | *PDGFRA CSRP3* |
| **Defense response to virus** | 0.0148 | 16.94 | 4 | 102 | *IFIH1 IFIT3 RSAD2 MX1* |
| **Response to virus** | 0.0194 | 12.71 | 4 | 136 | *IFIH1 IFIT3 RSAD2 MX1* |
| **Cytokine-mediated signaling pathway** | 0.0225 | 11.45 | 4 | 151 | *KRT8 KRT18 PARP14 MX1* |
| **Defense response to other organism** | 0.0205 | 8.03 | 5 | 269 | *IFIH1 IFIT3 RSAD2 PARP14 MX1* |
| **Response to cytokine** | 0.0148 | 8.00 | 6 | 324 | *KRT8 KRT18 IFIH1 IFIT3 PARP14 MX1* |
| **Cellular response to cytokine stimulus** | 0.0244 | 7.32 | 5 | 295 | *KRT8 KRT18 IFIT3 PARP14 MX1* |
| **Response to external biotic stimulus** | 0.0182 | 6.55 | 6 | 396 | *KRT8 IFIH1 IFIT3 RSAD2 PARP14 MX1* |
| **Response to other organism** | 0.0182 | 6.55 | 6 | 396 | *KRT8 IFIH1 IFIT3 RSAD2 PARP14 MX1* |
| **Immune response** | 0.0148 | 6.28 | 7 | 482 | *IFIH1 IFIT3 RSAD2 PARP14 CD36 JSP.1 MX1* |
| **Defense response** | 0.0198 | 6.26 | 6 | 414 | *IFIH1 IFIT3 CSRP3 RSAD2 PARP14 MX1* |
| **Biological proc. Involved in interspecies interaction**  **between organisms** | 0.0244 | 5.62 | 6 | 461 | *KRT8 IFIH1 IFIT3 RSAD2 PARP14 MX1* |
| **Response to external stimulus** | 0.0176 | 4.58 | 8 | 755 | *KRT8 PDGFRA IFIH1 IFIT3 CSRP3 RSAD2 PARP14 MX1* |
| **Cellular response to organic substance** | 0.0226 | 4.06 | 8 | 851 | *KRT8 KRT18 PDGFRA IFIH1 IFIT3 CSRP3 PARP14 MX1* |
| **Immune system proc.** | 0.0226 | 4.05 | 8 | 853 | *PDGFRA IFIH1 IFIT3 RSAD2 PARP14 CD36 JSP.1 MX1* |
| **Cell surface receptor signaling pathway** | 0.0314 | 3.78 | 8 | 914 | *KRT8 KRT18 DKK3 PDGFRA CSRP3 PARP14 CD36 MX1* |

**Supplementary Table 3.** **Melatonin Added** – Potential metabolic function of (all) genes differentially expressed in cumulus cells with the addition of melatonin to maturation medium (in the presence of complex proteins). (Blue down and red up in the presence of melatonin)

| **GeneName** | Metabolic function | Reference |
| --- | --- | --- |
| *DKK3* | Has been shown to alter glycolysis in pancreatic cancer BxPC-3 cells | (3) (Guo *et al.*  2021) |
| *GAL* | Galanin can regulate insulin release and is implicated in glucose metabolism (review) | (4) (Fang et al. 2020) |
| *PDGFRA* | Shown to mediate glycolysis through AKT activation in mouse glioma-derived tumor stem-like cells | (5) (Ran C, Liu H, Hitoshi Y & Israel MA 2013) |
| *PARP14* | IL-4 regulates glycolysis through PARP14 in some cancers (review) | (6) (Schweiker SS, Tauber AL, Sherry ME & Levonis SM 2018) |
| *AKAP12* | Gravin (coded by *Akap12*) has been shown to regulate lipid metabolism via *Srebp-2* in mouse liver | (7) (Fan et al. 2019) |
| *CSRP3* | *Csrp3*-KO mice show impaired glucose homeostasis in muscle in [8]; and reduced mitochondrial number and energy status of myocardium [9] | (8) (Hernandez-Carretero et al. 2018); (9) van den Bosch et al. 2005) |
| *KRT18/KRT8* | *KRT8* and *18* regulate glucose metabolism in H4-II-E-C3 hepatoma cell line (IL-6 induced) | (10) (Mathew et al. 2013) |
| *SGK1* | Involved in glucose metabolism and glucose/lipid homeostasis in multiple cell types and particularly cancer cells (review) | (11) (Sang et al. 2021) |
| *ODF2L* | IL-1R can interact with BCAP (encoded by *ODF2L*) to activate mTOR pathway and increase glycolysis in mouse Th17 cells. | (12) (Deason et al. 2018) |
| *CD36* | Aids in transport of fatty acids and regulates fatty acid metabolism in multiple cell types (review) | (13) (Silverstein RL & Febbraio M. 2009) |
| *ANXA6* | Shown to induce metabolic reprogramming between fatty acid oxidation and glycolysis in breast cancer cell lines | (14) (Williams SD & Sakwe AM. 2022) |
| *IGFBP5* | Shown to modulate lipid and glucose uptake in human hepatocyte (HepG2) cells and mouse in vivo model | (15) (Xiao Z, Chu Y & Qin W. 2020) |
| *IFIH1* | *IFIH1* codes for protein MDA5 which has been implicated as a metabolic switch regulating glycolysis in breast cancer cells | (16) (Zhang L et al. 2022) |
| *RSAD2* | Codes for viperin, which can interfere with mitochondrial beta oxidation in HEK293T, Human Foreskin Fibroblast, MEF cells and mouse adipocytes; also with cholesterol biosynthesis in HeLa and HEK293T cells (review) | (17) (Ghosh S & Marsh ENG. 2020) |
| *DSG2* | Mouse *Dsg2* knockout results in altered mTOR signalling reducing beta oxidation in cardiac myocytes leading to lipid accumulation. | (18) (Lin et al. 2023) |
| *GNAT3* | Gustducin (coded by *Gnat3*) regulates SGLT1 (glucose transporter – *Slc5a*) in mouse enteroendocrine cells | (19) (Margolskee et al. 2007) |

**Supplementary Table 4. Treatment specific methylated CpGs.** Number of genes within 1000 bases of relatively ‘hypermethylated’ CpG regions identified in a treatment specific manner (Figure 4), and selected “Biological Process” gene ontology (GO) terms.

| **Treatment** | **HypermethylatedGenes (No.)** | **GO - Biological Process (No.)** | **Genes** |
| --- | --- | --- | --- |
| **+CP-M** | 227 | Metabolic process  (54) | *ABAT, ACOXL, ACSF2, ADAMTS17, ALG1, ARRDC1, ASPHD1, CCNG2, CDC20, CHD1, CHST2, CLPS, CNTD2, COQ2, COQ5, COX10, CYP2J2, DCST1, DGAT1, DNAJC10, DPP3, EIF2B5, ETNK2, F13A1, F7, FEM1A, GALE, GALNT9, HS6ST1, HSP90B1, IKBKB, LDLRAP1, LIN9, MYLK4, NDUFA9, NDUFB8, NPHP3, NR1H3, PAPSS1, PIGZ, PRMT8, PSMD14, PTBP3, PTPA, RCHY1, ROR2, RPS6KA2, SERINC2, SMPD5, SRSF6, SSU72, TPP2, UFL1, YOD1* |
| **+CP+M** | 327 | Metabolic process  (82) | *ACER3, ACP1, ADRB3, AGPAT5, AIFM3, ALDH18A1, ASCL2, ASPHD1, CDK13, COL2A1, COQ2, CPOX, CTDP1, CYB5R3, DCK, DCLRE1C, DGKI, DPM1, DPM3, DPP3, DRAM2, DYRK1A, EHHADH, ELOVL7, EPOP, ETFB, FANCA, FOXL2, GAS6, GFOD1, GPC1, GPT2, HS6ST1, INSIG1, ISYNA1, LOC538435, MAPK7, MCM6, MMP15, MORF4L1, MRC2, MRPL17, MYH3, NADSYN1, NAPRT, NDUFAB1, NEK6, NR4A3, NRDE2, NTRK2, OAT, PARPBP, PATL1, PAWR, PAX6, PCSK1N, PES1, PGK1, PHETA2, PLK3, PNPLA4, POFUT2, PPCS, PPM1K, PPP4R1, PRKAA1, PRKCA, PSMC4, PTPN4, REV3L, SEC63, SETMAR, SIK3, SPTBN1, STYX, TBK1, TRIM62, TSEN54, TXNL1, UBQLN1, VCPIP1, ZDHHC6* |
|  |  | Negative regulation of biosynthetic process | *ASCL1, ASCL2, FOXL2, GAS6, GMNN, INSIG1, LBH, MSX2, MYPOP, NODAL, NR4A3, NR6A1, PAWR, PLK3, POU3F3, POU4F1, SIM2, TFAP4, WTIP, ZEB1* |
|  |  | Lipid biosynthetic process | *ACER3, COQ2, CYB5R3, DPM1, ELOVL7, INSIG1, ISYNA1, NDUFAB1, PRKAA1* |
| **-CP-M** | 250 | Metabolic process  (71) | *ABHD2, ACACA, ACER3, ADAM12, ADPRHL1, ADRB1, AK8, AUNIP, B3GAT1, CBX8, CRK, CTDSPL2, CTNS, CYP26A1, DAPK3, DIS3L, DSP, DUSP23, EFEMP1, EFL1, EPHB2, ERCC1, FBXO7, FOXO3, GAK, GALNT17, GAN, GBGT1, GUK1, HADHB, HOGA1, HOXB9, HSD17B10, INAVA, KCTD21, KLHL2, LRSAM1, MMP14, MTFR1, NAALAD2, NEURL2, NOVA2, NRDE2, NTRK2, P2RX7, PACSIN2, PAFAH1B2, PCSK2, PCSK5, PHKA2, PIGW, PLCD1, POLD1, POLE3, PRTN3, PTGDS, RCOR1, RTCA, SLC27A3, SOCS1, SPO11, SPSB4, SUMO2, TMEM150A, TSEN54, UBR5, URAD, USP4, VPS28, WSB1, ZFC3H1* |
|  |  | Catabolic process | *ABHD2, ACER3, CYP26A1, FBXO7, HADHB, HOGA1, KCTD21, LRSAM1, NAALAD2, P2RX7, PAFAH1B2, PLCD1, SPO11, URAD, USP4, VPS28* |
|  |  | Lipid metabolic process | *ABHD2, ACACA, ACER3, CRK, CYP26A1, HADHB, P2RX7, PAFAH1B2, PIGW, PLCD1, PTGDS, SLC27A3, TMEM150A* |
| **-CP+M** | 153 | Metabolic process  (36) | *ABHD17A, ADAM9, ADRB1, AGPAT5, ALYREF, APOH, ARIH2, ASB6, CCNT2, CHST2, CHST3, COMTD1, CRHBP, DICER1, FBXL17, FOXA1, GDF7, GLB1, GLUL, KLK9, MAN2C1, MBTPS1, MRC2, MYOD1, PER1, PLG, PPID, SDR42E2, SLX1A, SOX18, TOP2A, TRA2B, TUFM, URAD, WDR82, XXYLT1* |
|  |  | Response to lipid | *ADAM9, ADNP2, BCR, CRHBP, FOXA1, LRP6, MYOD1, PTGIR* |

**Supplementary Table References**

1. Ge SX, Jung D, Yao R. ShinyGO: a graphical gene-set enrichment tool for animals and plants. Bioinformatics. 2019;36(8):2628-9. doi: 10.1093/bioinformatics/btz931

2. Stelzer G, Rosen N, Plaschkes I, Zimmerman S, Twik M, Fishilevich S, et al. The GeneCards Suite: From Gene Data Mining to Disease Genome Sequence Analyses. Curr Protoc Bioinformatics. 2016;54:1.30.1-1..3. doi: 10.1002/cpbi.5

3. Guo Q, Chu Y, Li H, Shi D, Lin L, Lan W, et al. Dickkopf-related protein 3 alters aerobic glycolysis in pancreatic cancer BxPC-3 cells, promoting CD4(+) T-cell activation and function. Eur J Med Res. 2021;26(1):93. doi: 10.1186/s40001-021-00567-x

4. Fang P, Yu M, Shi M, Bo P, Zhang Z. Galanin peptide family regulation of glucose metabolism. Front Neuroendocrinol. 2020;56:100801. doi: 10.1016/j.yfrne.2019.100801

5. Ran C, Liu H, Hitoshi Y, Israel MA. Proliferation-independent control of tumor glycolysis by PDGFR-mediated AKT activation. Cancer Res. 2013;73(6):1831-43. doi: 10.1158/0008-5472.can-12-2460

6. Schweiker SS, Tauber AL, Sherry ME, Levonis SM. Structure, Function and Inhibition of Poly(ADP-ribose)polymerase, Member 14 (PARP14). Mini Rev Med Chem. 2018;18(19):1659-69. doi: 10.2174/1389557518666180816111749

7. Fan Q, Yin X, Rababa'h A, Diaz Diaz A, Wijaya CS, Singh S, et al. Absence of gravin-mediated signaling inhibits development of high-fat diet-induced hyperlipidemia and atherosclerosis. Am J Physiol Heart Circ Physiol. 2019;317(4):H793-h810. doi: 10.1152/ajpheart.00215.2019

8. Hernandez-Carretero A, Weber N, LaBarge SA, Peterka V, Doan NYT, Schenk S, et al. Cysteine- and glycine-rich protein 3 regulates glucose homeostasis in skeletal muscle. Am J Physiol Endocrinol Metab. 2018;315(2):E267-e78. doi: 10.1152/ajpendo.00435.2017

9. van den Bosch BJ, van den Burg CM, Schoonderwoerd K, Lindsey PJ, Scholte HR, de Coo RF, et al. Regional absence of mitochondria causing energy depletion in the myocardium of muscle LIM protein knockout mice. Cardiovasc Res. 2005;65(2):411-8. doi: 10.1016/j.cardiores.2004.10.025

10. Mathew J, Loranger A, Gilbert S, Faure R, Marceau N. Keratin 8/18 regulation of glucose metabolism in normal versus cancerous hepatic cells through differential modulation of hexokinase status and insulin signaling. Exp Cell Res. 2013;319(4):474-86. doi: 10.1016/j.yexcr.2012.11.011

11. Sang Y, Kong P, Zhang S, Zhang L, Cao Y, Duan X, et al. SGK1 in Human Cancer: Emerging Roles and Mechanisms. Front Oncol. 2020;10:608722. doi: 10.3389/fonc.2020.608722

12. Deason K, Troutman TD, Jain A, Challa DK, Mandraju R, Brewer T, et al. BCAP links IL-1R to the PI3K-mTOR pathway and regulates pathogenic Th17 cell differentiation. J Exp Med. 2018;215(9):2413-28. doi: 10.1084/jem.20171810

13. Silverstein RL, Febbraio M. CD36, a scavenger receptor involved in immunity, metabolism, angiogenesis, and behavior. Sci Signal. 2009;2(72):re3. doi: 10.1126/scisignal.272re3

14. Williams SD, Sakwe AM. Reduced Expression of Annexin A6 Induces Metabolic Reprogramming That Favors Rapid Fatty Acid Oxidation in Triple-Negative Breast Cancer Cells. Cancers (Basel). 2022;14(5). doi: 10.3390/cancers14051108

15. Xiao Z, Chu Y, Qin W. IGFBP5 modulates lipid metabolism and insulin sensitivity through activating AMPK pathway in non-alcoholic fatty liver disease. Life Sci. 2020;256:117997. doi: 10.1016/j.lfs.2020.117997

16. Zhang L, Hu X, Wu H, Tian H, Zeng J, Song D, et al. Knockdown of ARL5B Induces Mitochondrial-mediated Apoptosis and Inhibits Glycolysis in Breast Cancer Cells by Activating MDA5 Signaling. Curr Cancer Drug Targets. 2022;22(10):843-53. doi: 10.2174/1568009622666220511112538

17. Ghosh S, Marsh ENG. Viperin: An ancient radical SAM enzyme finds its place in modern cellular metabolism and innate immunity. J Biol Chem. 2020;295(33):11513-28. doi: 10.1074/jbc.REV120.012784

18. Lin Y, Liu R, Huang Y, Yang Z, Xian J, Huang J, et al. Reactivation of PPARα alleviates myocardial lipid accumulation and cardiac dysfunction by improving fatty acid β-oxidation in Dsg2-deficient arrhythmogenic cardiomyopathy. Acta Pharm Sin B. 2023;13(1):192-203. doi: 10.1016/j.apsb.2022.05.018

19. Margolskee RF, Dyer J, Kokrashvili Z, Salmon KS, Ilegems E, Daly K, et al. T1R3 and gustducin in gut sense sugars to regulate expression of Na+-glucose cotransporter 1. Proc Natl Acad Sci U S A. 2007;104(38):15075-80. doi: 10.1073/pnas.0706678104
